# Aspergillosis, Avian Species and the One Health Perspective: The Possible Importance of Birds in Azole Resistance

**DOI:** 10.3390/microorganisms8122037

**Published:** 2020-12-19

**Authors:** Aryse Martins Melo, David A. Stevens, Lisa A. Tell, Cristina Veríssimo, Raquel Sabino, Melissa Orzechowski Xavier

**Affiliations:** 1 Programa de Pós-Graduação em Microbiologia e Parasitologia, Instituto de Biologia, Universidade Federal de Pelotas, Pelotas 96160-000, Brazil; melissaxavierfurg@gmail.com; 2 Reference Unit for Parasitic and Fungal Infections, Department of Infectious Diseases, National Institute of Health, Dr. Ricardo Jorge, 1649-016 Lisbon, Portugal; cristina.verissimo@insa.min-saude.pt (C.V.); raquel.sabino@insa.min-saude.pt (R.S.); 3 Division of Infectious Diseases and Geographic Medicine, Stanford University Medical School, Stanford, CA 94305, USA; stevens@stanford.edu; 4 California Institute for Medical Research, San Jose, CA 95128, USA; 5 Department of Medicine and Epidemiology, School of Veterinary Medicine, University of California, Davis, CA 95616, USA; latell@ucdavis.edu; 6 Instituto de Saúde Ambiental, Faculdade de Medicina, Universidade de Lisboa, 1649-028 Lisboa, Portugal; 7 Programa de Pós-Graduação em Ciências da Saúde, Faculdade de Medicina, Universidade Federal do Rio Grande, Rio Grande 96203-900, Brazil

**Keywords:** *Aspergillus*, azole resistance, avian aspergillosis, invasive aspergillosis, One Health context

## Abstract

The One Health context considers health based on three pillars: humans, animals, and environment. This approach is a strong ally in the surveillance of infectious diseases and in the development of prevention strategies. *Aspergillus* spp. are fungi that fit substantially in this context, in view of their ubiquity, as well as their importance as plant pathogens, and potentially fatal pathogens for, particularly, humans and avian species. In addition, the emergence of azole resistance, mainly in *Aspergillus fumigatus* sensu stricto, and the proven role of fungicides widely used on crops, reinforces the need for a multidisciplinary approach to this problem. Avian species are involved in short and long distance travel between different types of landscapes, such as agricultural fields, natural environments and urban environments. Thus, birds can play an important role in the dispersion of *Aspergillus*, and of special concern, azole-resistant strains. In addition, some bird species are particularly susceptible to aspergillosis. Therefore, avian aspergillosis could be considered as an environmental health indicator. In this review, aspergillosis in humans and birds will be discussed, with focus on the presence of *Aspergillus* in the environment. We will relate these issues with the emergence of azole resistance on *Aspergillus*. These topics will be therefore considered and reviewed from the “One Health” perspective.

## 1. Introduction

**Figure 1:**
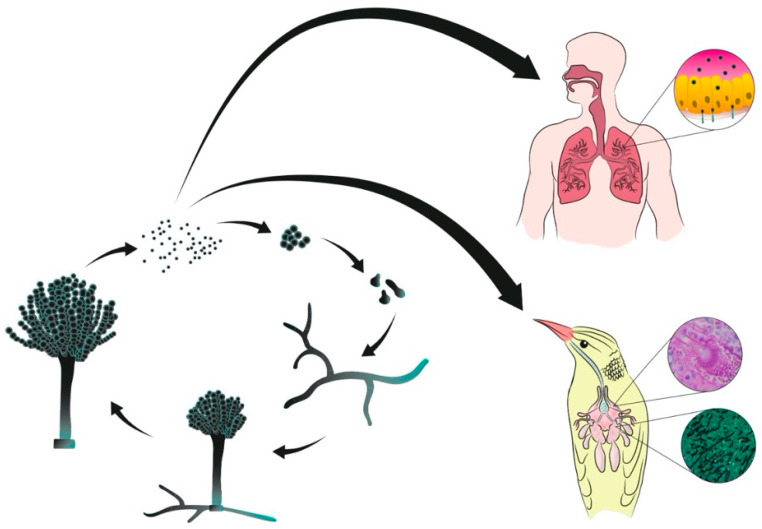
Representation of vegetative cycle of *Aspergillus* spp. and infection of human and avian hosts by inhalation of conidia.

Aspergillosis is a fungal disease caused by *Aspergillus*, which can affect humans, dogs, cats, horses, marine mammals, wild and domestic birds and even invertebrates, such as bees and corals [1]. The common route of infection for vertebrates is inhalation of conidia present in the environment and the respiratory tract is the most common anatomical site for initial site of infection (Figure 1) [1,2]. To date, aspergillosis is considered a non-contagious disease, however recent studies have raised the possibility of transmission of this fungus among hospitalized patients [3,4]. In this sense, due to its clinical manifestation in avian species [5,6], the hypothesis of fungal transmission among captive birds, as in the case of aviaries, wildlife rehabilitation centers and zoological institutions must be raised. This transmission may occur especially through environmental (particularly air) contamination by sick birds.

Azoles such as itraconazole (ITC), voriconazole (VRC), posaconazole (POS) and isavuconazole (ISA) are the drugs of choice for prophylaxis and treatment of aspergillosis both in humans and animals [7,8,9,10,11,12]. Because of the difficulty on designing antifungal drugs that lack side effects in humans, effective therapeutic options to treat mycoses presently are limited [13]. Concurrently, triazoles are the main pesticides used in agriculture [14,15]. Thus, a concern about worldwide azole resistance arises from the large use of agricultural fungicides in the environment, leading to emerging resistant *Aspergillus* strains, and hence contributing to an increase of treatment failure rate in humans.

In this review, we will discuss relevant microbiologic aspects of these fungi, and of its infection, aspergillosis, in humans and birds. Aspects concerning *Aspergillus* in the environment, and the emergence of antifungal resistance of this pathogen will also be approached. We highlight these topics from a One Health perspective, where humans, animals and environment are all connected.

## 2. *Aspergillus*

*Aspergillus* is a ubiquitous saprophytic genus, with worldwide distribution. It is frequently found in decomposing plants, playing an important role in this process and contributing to carbon and nitrogen recycling [16]. Fungi of this genus are widely used in industry owing to their high capability to produce a diversity of enzymes, such as amylases; in generating chemical additives, such as citric acid; the production of soy sauces; and in bioremediation processes, among others [17].

The taxonomy of *Aspergillus* is in flux, and four to eight subgenera, and from 16 to 25 sections, has been proposed by different authors, and more than 350 species. Most species are found in the environment, without reported clinical relevance thus far [18,19,20,21]. Sections *Flavi*, *Nigri*, *Nidulantes, Terrei* and *Fumigati* are of interest in clinical practice. 

Species belonging to section *Fumigati* are the main etiologic agents of aspergillosis. *Aspergillus fumigatus* sensu stricto is the cause of the majority of those infections. However, it is estimated that between 3 to 6% of those infections are caused by cryptic species. *A. lentulus, A. udagawae, A. viridinutans, A. thermomutatus, A. novofumigatus* and *A. hiratsukae* are the most common cryptic species reported in medical practice to date. When compared to *A. fumigatus* sensu stricto, these cryptic species seem to have limited pathogenicity, since they have limited thermotolerance and have slower rates of production of different mycotoxins. On the other hand, most of these species have intrinsic resistance to azoles, which makes them refractory to the treatment of choice for aspergillosis [22,23,24,25].

Some characteristics present in *Aspergillus fumigatus* sensu stricto can partially explain why this species is the leading human and animal pathogen, such as rapid growth, small size of the conidia (1–4 µm), thermotolerance (growth between 15 °C and 55 °C, able to tolerate up to 70 °C), tolerance to high pH, low nutritional requirements, and production of secondary metabolites such as gliotoxin. The latter represents an important virulence factor [16,22,23,26] helping in tissue invasion. High thermotolerance is a factor that could benefit *A. fumigatus* in the environment, in comparison to other fungal species: its optimal growth temperature is 37 °C [27], which allows this species to be an important human pathogen. Given the current conditions of global warming, this feature could favor *A. fumigatus* growth and its dispersion in the environment [28].

## 3. Aspergillosis in Humans

According to the Global Action Fund for Fungal Infections (GAFFI), it is estimated that the health of more than 15 million people is affected by aspergillosis, causing more than 1 million deaths per year [29]. As fungal infections increase in clinical medicine [30,31,32], the number of cases of aspergillosis is likewise increasing through the past decades. An epidemiological study performed in the United States suggests an increase from 3 cases of invasive aspergillosis (IA) per 10,000 people admitted for hospital care (data obtained in 1996) to 10 cases per 10,000 (data obtained between 2009 and 2013) [33].

The clinical manifestations of aspergillosis in humans are classified according to the extent of mycelial colonization or tissue invasion and are influenced by the host’s immune response capacity. The range of consequences includes allergic reactions and invasive infections. Patients with co-morbidities such as chronic granulomatous disease, bone marrow or solid organ transplantation, prolonged neutropenia, AIDS, or with prolonged treatment with steroids or other immunosuppressive drugs are risk groups for IA [8,32], and mortality rates due to acute IA can reach 80% in the first year after diagnosis [34,35]. Recent studies emphasize the importance of IA in intensive care units, with impressive increase in numbers over the past few decades, in which mortality rates can reach 100% [36] especially in patients with influenza, in patients receiving newer cancer chemotherapy modalities, and in patients diagnosed with COVID-19 [37,38,39,40,41,42]. 

*Aspergilli* in the section *Fumigati* are the most common etiological agents in all the aspergillosis presentations described above. The species *A. fumigatus* sensu stricto is the most frequently isolated agent of aspergillosis in humans. Since the beginning of the 2000s, however, *Aspergillus* sections have been studied more intensively at the molecular level, and cryptic species of the *Fumigati* section, such as *A. lentulus*, *A. thermomutatus*, and species of the *viridinutans* complex such as *A. pseudofischeri* and *A. felis,* among others, have also been described in aspergillosis cases [22,43,44,45,46].

Aspergillosis cases have been classified according to the guidelines of the European Organization for Research and Treatment of Cancer/Invasive Fungal Infections Cooperative Group and the National Institute of Allergy and Infectious Diseases Mycoses Study Group (EORTC/MSG), [47]. However, with the increasing knowledge of new risk groups and with different characteristics, aspergillosis case definitions may change, according to the analyzed group as well as the clinical manifestations. Case definitions specifically for chronic pulmonary aspergillosis [48] and for influenza-associated pulmonary aspergillosis in intensive care unit patients [49] have been described. Also, given some differences already found in cases of Covid-associated pulmonary aspergillosis (CAPA), epidemiological studies are being performed to support the case definition of CAPA [42,49,50].

## 4. Aspergillosis in Avian Species

Birds are especially susceptible to aspergillosis because of their anatomical and physiological characteristics, including the presence of air sacs with poor vascularization and limited mucociliary function, providing an ideal environment for fungal growth. Additionally, birds have heterophils instead of neutrophils, which could be less effective against hyphal invasion [6,51,52]. Aspergillosis is therefore a major cause of morbidity and mortality in birds, causing economic and ecological damages. Furthermore, this disease can be responsible for impacting avian species in zoological institutions and in wild animals during rehabilitation [51,53,54,55].

*Aspergillus* section *Fumigati* is responsible for up to 90% of deaths in birds with aspergillosis [12,52,53]. *A. fumigatus* sensu stricto is the only etiologic agent of this section identified in birds so far [55,56,57]. This may be related to the virulence traits of this species: the ability to grow at higher temperatures, such as the body temperature of birds (38–42 °C); the reduced size of the conidia in comparison with other sections of the genus, facilitating penetration into the lower respiratory tract; and the enhanced production of gliotoxin [23,53,58].

**Figure 2:**
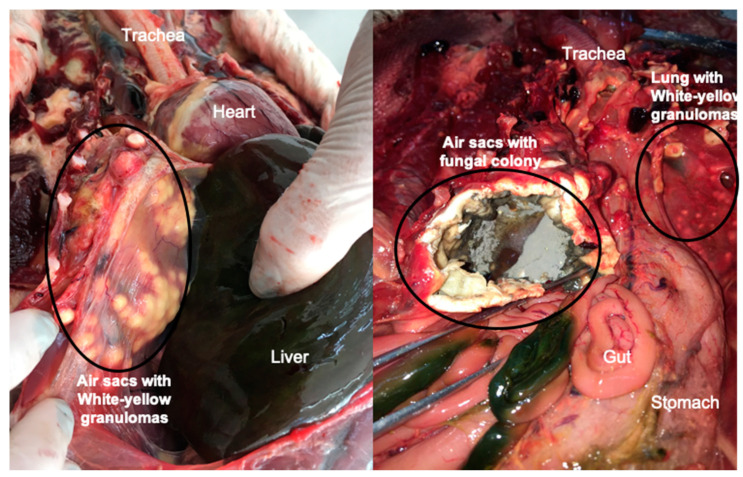
Common lesions of pulmonary aspergillosis found in avian species A: White-yellow granulomas on air sac membranes from a Magellanic penguin that died during rehabilitation at the Center of Rehabilitation of Marine Animals, Brazil, B: Growth of fungal colonies of *Aspergillus fumigatus* on air sac membranes with evidence of substantial sporulation, from a free-ranging Magellanic penguin, found dead on Cassino beach, Rio Grande, Brazil.

The clinical signs of aspergillosis in birds are nonspecific and often not evident until the final stage of the disease. For certain avian species, such as penguins and albatrosses being treated in rehabilitation centers [12,55], and poultry (i.e., turkeys and chicks) in production settings [51,59] the management of flocks is more common than an individual bird, and therefore an earlier diagnosis can be an even bigger challenge. In this context, early diagnosis is essential for determining treatments, and serological assays seems to be the most promising so far [59,60,61,62,63,64,65]. However, since an efficient and financially accessible method for making an early diagnosis is not available, the diagnosis of aspergillosis in birds is commonly made only on *post-mortem* examination. Macroscopic findings during necropsy are described as white-yellow granulomas in pulmonary parenchyma and/or air sac membranes, and in some cases the dissemination of the disease to other organs, such as heart, liver, kidneys and spleen. In addition, the growth of fungal colonies, with moderate to large production of conidia in the lower respiratory tract can occur, and it has been reported in several avian species [5,6,51,66,67] (Figure 2). Confirmation of the diagnosis is made by isolation of the fungus in culture associated with evidence of tissue invasion, and observation of hyphae in histopathology [6,53]. 

Aspergillosis is reported in poultry production and in different types of avian livestock, such as chickens, turkeys, geese, ducks, pigeons, emus and ostriches. Young birds are the most affected [59,68,69,70]. It is common to observe outbreaks of aspergillosis in poultry, with mortality rates varying between 4.5 and 90% [51]. The economic losses in this group can reach US$ 11 million per year in turkey production [66]. The losses are related to the condemnation of carcasses in slaughterhouses owing to air sacculitis and fungal pneumonia, reduction of the growth rate of the birds, and increase of the mortality rate [1,51,69]. The risk factors for poultry include high environmental humidity, lack of well-ventilated housing, accumulation of organic matter and use of bedding litter made rich with organic matter [51,71,72]. These environmental conditions are optimal for *Aspergillus* growth. High humidity levels and richness of organic matter are factors that contribute to fungal multiplication and more airborne conidia, consequently enhancing the chance of inhaling a larger inoculum, one of the risk factors for the development of avian aspergillosis [12,16,51,52,54].

Aspergillosis is also a concern given the high mortality of wild birds kept in captivity, such as zoological institutions, avian wildlife rehabilitation centers, and captive birds of prey used for falconry [54,55,56,73,74]. In wildlife rehabilitation centers, aspergillosis is considered an important fungal disease for penguins in rehabilitation [54], with a mortality rate of ~50% in Magellanic penguins (*Spheniscus magellanicus*) in captivity [12]. In zoological institutions, in addition to the occurrence of *Aspergillus* outbreaks, deaths caused by aspergillosis are common [75,76,77]. There are many knowledge gaps regarding free-ranging wild birds, although some reports have been published regarding the frequency of aspergillosis, in comparison to what is known regarding aspergillosis in captive birds [78,79,80]. 

Wild birds brought into captivity are commonly administered azoles, for both prophylaxis and treatment, given the high incidence of fungal diseases in rehabilitation centers and the potential for increased exposure [9,11,12,55,81]. Captive populations of birds in zoological institutions are also prophylactically treated and this can be related to species susceptibility and environmental challenges. Regarding poultry farms, the use of azoles for prophylaxis and treatment of aspergillosis is unusual, owing to concerns regarding the existence of drug residues in meat and the expense. However, unusual circumstances could lead to antifungal prophylaxis on farms [82]. On the other hand, the use of azoles for decontaminating bedding and environmental disinfection is common [51,70,71,82,83,84,85].

## 5. *Aspergillus* Azole Resistance

High values of antifungal minimal inhibitory concentrations against different *Aspergillus* sections and intrinsic antifungal resistance described for several cryptic species of *Aspergillus* isolated from environmental sources has been described. This highlights the importance of establishing surveillance programs for *Aspergillus* antifungal susceptibility from different sources [86].

The increase in azole resistance described for *A. fumigatus* sensu stricto is a global concern [45,87,88]. The first report of azole resistance in clinical isolates was published in 1997 [89]. Since then, many reports of clinical and environmental azole resistant isolates from different regions of the world have been reported [87,89,90,91,92,93,94,95,96]. A retrospective/prospective study in a laboratory of the United Kingdom has shown that the resistance rate of *A. fumigatus* has increased from 0.43 (1998–2011) to 2.2% (2015–2017) [97], and another study from Denmark has shown an increase from 1.4 to 6% in azole resistance from human clinical isolates [98]. 

The emergence of azole resistance in *A. fumigatus* sensu stricto deserves special attention, since this is the most common species of *Aspergillus* related to human and animal aspergillosis, as stated. In addition, given the limited availability of antifungal drugs to treat fungal infections, this increase in resistance rates found in clinical practice poses a substantial problem and could result in treatment failure and a consequent increase in mortality rates [99]. Current local azole resistance rates vary between 0 and 26%, variation occurring according to geographic region and patient population [86,91,97,100,101,102,103,104,105,106]. An investigation, involving 13 countries in four continents, found an azole resistance rate of 6% among 2026 *Aspergillus* isolates evaluated [107].

Regarding avian species, despite the importance of aspergillosis in these animals, there are few studies evaluating resistance or cryptic species. The majority of those reports are in poultry, and the rate of resistance is considered low thus far [70,82,108,109]. On the other hand, a few reports on *Aspergillus* susceptibility studies isolated from captive wild birds are described [110,111]. Studies on aspergillosis in free-ranging wild birds are scarce, and consequently, knowledge about epidemiological trends [78,79,80] is insufficient, and susceptibility assays on *Aspergillus* isolated from this group of birds have not been published so far.

## 6. Main Mechanisms of Azole Resistance

Azole drugs act directly by inhibiting ergosterol biosynthesis, binding to the enzyme 14α-lanosterol demethylase (CYP51), preventing the conversion of lanosterol to ergosterol [88,91]. The *cyp51A* gene is directly related to the emergence of *A. fumigatus* sensu stricto azole resistance [87,91,112]. 

Long-term treatments, such as those carried out in cystic fibrosis patients, are an important route to azole resistance, and single nucleotide polymorphisms (SNP) which result in amino acids changes such as G54, G138, M220, and G448, among others, are more commonly found in these patients [113]. Point mutations as G54 and M220 can change the protein structure, which affects the docking of certain azole compounds for the whole protein [114,115,116]. On the other hand, mutations such as TR_34_/L98H, which confers pan-azole resistance [100,101], and TR_46_/Y121F/T289A, apparently more related to a high level of voriconazole resistance and variable susceptibility to itraconazole [114,117,118], are reported in environmental strains [101,119], in patients with long-term treatment as well as in patients with IA [90,106,120,121]. 

Overexpression of *cyp51* is another mechanism associated with resistance in *A. fumigatus* [88,113]. Despite the great importance of *cyp51A* mutations conferring azole resistance, a survey of Manchester isolates showed that 43% of resistant isolates were not *cyp51A* mutants [122]. Thus other mechanisms than *cyp51A* mutation may have an important role in azole resistance, such as point mutation in the subunit HapE, of the CCAAT-biding complex (CBC) [123,124], in *cox10* gene [125], and in *hmg1* gene [126]. In this sense, genome-wide sequencing is a good tool to identify possible mutations conferring azole resistance in *cyp51*-unrelated resistant strains [125,126].

The overexpression of efflux pumps has already been proven in resistant strains without changes in the *cyp51* gene [127,128], such as overexpression of genes that encodes proteins from the ATP-binding cassette of the ABC transporters class [128,129,130,131,132], as well as from the major facilitator transporter (MFS transporter) [133,134].

The production of an extracellular matrix (ECM) by *A. fumigatus*, in biofilm formation, plays a significant role in antifungal resistance, shielding the fungus from the drugs and reducing antifungal susceptibility [135,136]. In addition, the overexpression of efflux pumps in association with biofilm formation has been related to azole resistance [137]. More studies aiming to understand the real importance of biofilm formation and its contribution to the emergence of azole resistance are necessary. 

## 7. The Role of Pesticides in Emerging *Aspergillus* Azole Resistance

Treatment failures in IA patients have raised the discussion of the origin of resistance, since many of those patients had never been under previous azole therapy. Environmental exposure is the most reasonable possibility as the source of resistant strains [138]. In this context, investigations regarding possible sources of resistance for environmental strains were initiated and the large use of azole fungicides in agriculture was identified as a probable factor in the emergence of resistant strains in the environment [139]. 

This hypothesis was later supported by numerous other findings, such as the predominance of two mutations (TR_34_/L98H and TR_46_/Y121F/T298A) in the majority of clinical isolates from different care centers, especially in azole-naïve patients [87,121,140,141]. The presence of the same mutation in environmental isolates [92,101,142], the genetic similarity between clinical and environmental isolates [118,138,139,143], the global spread of these mutations [92,94,96,101,144,145] and most recently, studies showing the lower diversity present in resistant strains with these mutations in relation to wild type strains [107,146] support this hypothesis. Since then, several studies have shown the role of azole pesticides used in agriculture in the selection of resistant strains of *A. fumigatus* [92,117,142,147,148].

Practices to control infestations in crops are essential, given that plant pathogens, particularly fungi, can cause huge economic losses in many agricultural endeavors, such as the culture of grapes [149], rice (an average of US$ 69.34 million lost annually in US [150]), soybeans [151], corn [152], among many others. It is estimated that such global losses in the five more important crops occur on a scale that, if mitigated, would be enough to feed 8.5% of 7 billion people in 2011 [153]. There has been controversy over pesticides that could lead to resistance. Their ban is advocated by several scientists. In this sense, the list of permitted pesticides in each country is modified periodically by decision of the government based on available studies, and thus, it varies greatly between different countries. Among the pest control mechanisms, azole pesticides are currently widely used on crops, such as bromucazole, cyproconazole, diphenoconazole, epoxiconazole, fluquiconazole, flutriafol, imibenconazole, ipconazole, metconazole, myclobutanil, propiconazole, tebuconazole, tetraxonazole and triticonazole [154,155]. The control of *Aspergillus* in cereal crops is important mainly because of the mycotoxin production by some species. Aflatoxins, which are produced especially by *A. flavus*, represent a high risk to human and animal health, with serious implications such as hepatotoxicity, teratogenicity and immunotoxicity [156]. Ochratoxin A, first described in *A. ochraceus*, and produced by several species of *Aspergillus*, with nephrotoxic and genotoxic effects, is a probable cause of cancer for humans [157]. *A. fumigatus* is not the main target of fungicides used in crops. However, since *Aspergillus* fungi are saprophytic and ubiquitous, the presence of its spores in these areas is common. The use of azole fungicides culminates in the selection of resistant strains, which then increase in the environment [146]. 

Many of these crop fungicides have molecular similarity with azoles used in clinical settings, sharing as the target, ergosterol biosynthesis [142]. Because of the same action target and the structural similarity of the molecules, the azole crop fungicides can lead to cross resistance with azole drugs available to treat aspergillosis in clinical cases [138]. Thus the search for alternative options to the use of fungicides for pest control in crops is urgently needed and, concurrently, the search for more effective drug options to treat fungal diseases [158].

## 8. Contamination of the Environment with Pesticides

The fungicide-driven route to the emergence of azole resistance in *A. fumigatus* is already a well-described fact. However, analyzing this issue in a broader ecological context, this problem spreads beyond the limits of the lands where those chemicals are used. Azoles fungicides are considered pesticides with high chemical and photochemical stability, low biodegradability and are easily transported into the environment, making them persistent in soil and water [159]. Fruits, vegetables and flowers cultured in farms that regularly use fungicides enter in thousands of homes daily. In this route, they can carry traces of those chemicals with them inside our homes [155]. In addition, there is contamination of drinking and irrigation water, and adjacent soils with these chemicals, mainly drained by rainfall [160]. It is true that the rains also end up diluting these fungicides; however, if they are in sub-inhibitory doses, this can also contribute to the emergence of resistance, such as by stimulating the overexpression of efflux pump genes [161].

Unfortunately, contamination of different environments by crop fungicides occurs worldwide. In this context, some countries have laws to determine the maximum acceptable concentrations of these products in both water and food, but in other countries, mainly those in development, there is no regulation of these limits yet. In Cameroon, where there is no government regulation, fungicides such as metalaxyl, carbendazim, tetraconazole and penconazole are found in between 22 and 100% of sampled surface waters from the Méfou watershed [160]. In Spain, a study regarding the concentration of new-generation fungicides released from crops in throughfall (the rainfall that is not arrested in the plant canopy) were determined during rainfall episodes, and concluded that these concentrations far exceeded the maximum permissible levels for drinking water established by the European Union (EU) regulations. Although this throughfall water is not used directly for drinking, it contaminates soil, runoff and water courses, and concentrations of those fungicides could exceed the limits established by the EU [162]. In areas of broad use of fungicides in the USA, at least two fungicides were detected in 55% of bed sediments and 83% of suspended solid, sampled from three different geographic areas, showing that these chemicals can persist in the environment in variable concentrations [163]. Again, in a USA study, during a heavy fungicide application period, azole fungicides such as propiconazole and metconazole, in addition to other classes of fungicides, were found in different types of wetlands located in and near those fields [164]. In an important region for rice growing in Brazil, high levels of tebuconazole (up to 460 ng·L^−1^) were found in surface and drinking waters [165].

## 9. Use of Crop Areas and Adjacent Areas by Birds

The presence of resident and migratory birds in plantation areas and their surroundings is very common. These areas serve both as feeding and resting grounds for many avian species, in large part due to the loss of their natural habitats, such as natural wetlands, on which waterbird species depend throughout their life cycle [166]. Most commonly, the presence of birds in cultivation areas results in conflict, with economic losses in agriculture, such as the case of wine grapes in single vineyards [167]. However, in some cases, this relationship can be beneficial for both sides, as for sustainable management in some rice fields in the Mediterranean area, where migratory waterbirds forage in rice fields, and farmers benefit from the nutrient enrichment of the soils due to the defecation of these birds [168].

In Europe, a total of 121 avian species were observed, at least occasionally, in rice fields; many of them using these fields to forage and/or breed [169]. In the Americas, 169 waterbird species and 166 landbird species were recorded in rice paddies [170]. In Asia, 135 different bird species were noted using Japanese and Korean rice paddies [171]; in India, at least 351 species [172] were counted in those areas. In West Africa, the density of shorebirds in shallow water is 4–6/hectare and of the waterbirds is 11/hectare in wet coastal rice fields [173]. In wine grape vineyards, the presence of birds in crop areas is also well recorded, such as in two farms of Canada, where 8 species and more than 1.000 individuals were recorded, many with foraging behavior [167]. Another study in the USA reported the presence of 29 fruit-eating avian species in different fruit crops, such as grapes, apples, blueberries and sweet cherries, some of them in large numbers [174]. Hence, a high diversity of birds (carnivorous, herbivorous, insectivorous and omnivorous) tend to be found in these areas. Many migratory birds use these areas as stop-over sites as well, such as the use of cover crops in the US Midwest Corn Belt region during spring migration [175]. In addition, flower crops, such as tulips, are important areas used by some birds, mainly passerines (perching birds), during some phases of their life-cycle [176,177].

The movements of avian species between these areas can vary according to season, migration, and rainfall patterns; and may or may not coincide with seasons of pesticide use. However, the direct interaction of birds with seasons of pesticide use is not a crucial factor for our analyses, since the main subject of our interest is the interaction of birds with azole-resistant *A. fumigatus* isolates selected due to the use of agricultural fungicides. These chemicals remain in the environment for a long time. In this sense, any birds that have contact with these lands and adjacent areas at any time may be exposed and colonized or infected by azole-resistant fungal strains. Estuaries and adjacent waterways are natural habitats for a high diversity of endemic avian species such as passerines, waterbirds and shorebirds, as well as migratory birds [178]. As previously discussed, these areas are directly impacted by fungicide contamination, which can remain in the environment for extended periods. This is a potential route of exposure to azole resistant strains. The contamination of these environments can also occur through an alternative route: the migratory routes of birds is a potentially important route for the dispersion of resistant strains [166,179], as we will discuss next.

## 10. Bird Migration and Their Role in Pathogen Dispersion

The importance of migratory birds in the spread of pathogens such as viruses, bacteria and fungi that can affect not only other animals, but also humans, is already known [180]. Pathogenic bacteria were isolated from fecal and blood samples of migratory birds captured along the Mediterranean and Black Sea, and some of these microorganisms were shown to be multi-drug resistant [179]. Perhaps the most well-known role of avian species in microorganism dispersion is with viruses [181,182,183], and genomic diversity studies have proven the spread of viruses through bird migration, as well as the potential risk of transmission from wild birds to poultry and vice versa [184]. 

In addition, a genomic study demonstrated that gulls are a reservoir of *Candida glabrata,* including fluconazole-resistant strains, and they can facilitate the spread of this microorganism and promote the indirect transmission to humans via environmental contamination [185]. Recently, the role of birds in the dispersion of the highly pathogenic multi-drug resistant yeast, *Candida auris,* was also proposed [186]. 

During migration, birds are exposed to several stressors, such as weight loss, fatigue, food deprivation, among others [187], which can be predisposing factors to infectious diseases, such as aspergillosis. They can become infected by the inhalation of conidia during foraging, or even during rest time. Since avian species can be considered as a health indicator of a certain ecosystem [188], the surveillance of aspergillosis in wild birds could be an important tool to better understand environmental azole resistance, particularly its geographic distribution. We may anticipate threats to human health defined in such studies, such as the introduction of *Aspergillus* isolates with new resistance profiles, the emergence of new resistance mechanisms and an increase in the rate of resistance in various regions.

Moreover, direct and/or indirect transmission of *Aspergillus* strains from wild birds to poultry and humans should not be disregarded, particularly since reproductive structures of the fungus have been found on air sac membranes of birds with aspergillosis [5,6,78]. However, given a bird’s lack of a diaphragm and a negative pressure system in their thoracic cavity, it is unlikely that the birds can release conidia during respiration. Therefore, the more likely route of infection would be through their feces or contaminated carcasses that then contaminate the environment. Birds and humans share common geographic areas in rural areas (where many aviaries are found), but also urban and suburban areas, since most of the human population is located near water sources (rivers, estuaries, lagoons and the sea), essential environments throughout the life cycle of birds [189].

## 11. Why Should We Consider the Role of Birds in Azole Resistance in the One Health Approach?

The One Health context considers health in several dimensions, addressing the health of humans, animals, and finally, the health of the ecosystem [190]. In the One Health approach, the role of environmental changes in the emergence of chronic and infectious diseases is highlighted. The concept is supported in the development of disease prevention strategies by several international agencies, such as the Food and Agriculture Organization of the United Nations, the World Organization for Animal Health and the World Health Organization [191]. 

This approach had been essential to better understand the role of crop fungicides in the emergence of azole resistance in *A. fumigatus*, with consequent treatment failure and mortality especially in humans with IA [99,118]. Thus, multidisciplinary studies, involving the three pillars of the One Health concept, should be considered in an attempt to circumvent this emerging problem, bringing to light new solutions for the control of agricultural pests, for the control of environment in, e.g., poultry production, and for the prophylaxis and antifungal therapy in human and veterinary medicine [192,193].

A recent study showed the low diversity of azole resistant strains harboring the TR_34_/L98H and TR_46_/Y121F/T289A mutations, when compared with wild strains. The global distribution of those clonal *A. fumigatus* isolates, harboring these mutations, demonstrates the ability of these strains to spread throughout the world [146]. Another study showed the gene flow among regional and global populations, with the same genotypes found in countries up to 7500 km apart, suggesting intermediate to short-distance dispersals and long distance dispersals as credible patterns of spread of *A. fumigatus* [107]. 

**Figure 3:**
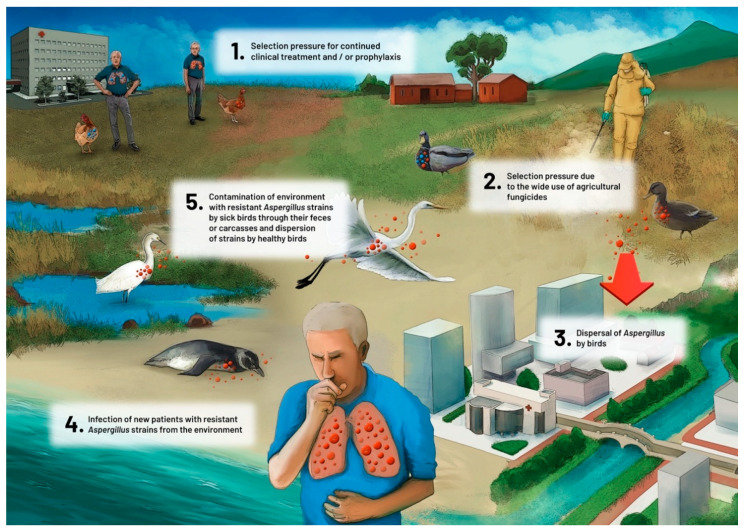
Proposed role of avian species in the dispersion of azole-resistant *Aspergillus fumigatus.*

Environmental dispersion by winds or dispersion by human population movements can be sources of dissemination. However, given the movements that birds routinely perform during their life cycle, with short to medium distance displacements for food and rest, and long distance displacements for seasonal migrations, the role of birds as contributors to dispersion of *Aspergillus* strains (such as those carrying specific mutations that confer azole resistance) should be considered. Some avian species are highly susceptible to aspergillosis, presenting large fungal colonies on their air sac membranes. By analogy, these birds resemble in vivo cultures of *Aspergillus*, with high fungal growth and high levels of sporulation of the fungus. The mortality among these birds is very high. Contamination of other birds and of the environment with resistant *Aspergillus* strains occurs through the feces of sick birds or carcasses and dispersion of strains by healthy birds (in their feathers, for example) (Figure 3). In this sense, the surveillance of mortality in wild birds and the identification of this disease in these animals could be an important indicator of environmental health, and a tool to monitor the emergence of new mechanisms of azole resistance that could have implications for human health.

**Figure 4:**
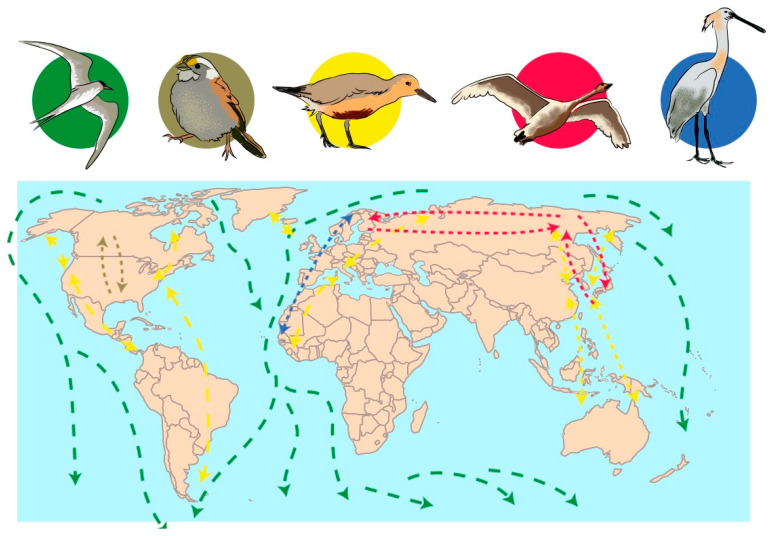
Simplified geographical examples of migratory routes of birds: In green, *Sterna paradisaea*; in brown, *Zonotrichia albicollis*; in yellow, *Calidris canutus*; in red, *Cygnus cygnus*; in blue, *Platalea leucorodia.* This figure was built based on information that is more detailed at https://www.birdlife.org.

Birds can carry pathogens without being sick themselves. The transportation of pathogens from one crop field to another or to nature can occurs through their digestive tract (beak, gut microbiota and feces), legs, and feathers [180,185,194,195]. This dispersion can occur over short distances, such as from a foraging field to where the birds rest, or over large distances, as in the case of migratory birds (Figure 4) [166,170,179,180]. For example, some birds such as *Calidris alba* (sanderling) migrate every year from the Artic region (where they breed in northern summer) to the southern hemisphere (where they feed during the northern winter), traversing from 3000 to 10,000 km twice a year. During this travel, they require some stop-overs to feed and rest [196]. This is an important example to show how far birds could transport strains of *Aspergillus* and the significant impact of the dispersion.

Another point still scarcely investigated so far, is the consequence for the emergence of azole resistance given the routine use of these drugs as prophylactic therapy in rehabilitation of wild birds, and the use of azole-based chemicals for disinfecting poultry houses [11,12,51,82,197,198]. In the case of azole prophylaxis in avian species, or the use of azole-based chemicals in poultry farms, these exposures could contribute to azole resistance development.

Regarding human exposure to birds, beyond zoological institutions, rehabilitation centers, farm and poultry production workers, and waterfowl hunters, it is important to highlight that the marketing of live wild birds, as well as poultry, in urban markets is a tradition in several countries, most of them in Far-East countries [199]. This tradition consequently increases the chance of people having contact with *Aspergillus* strains through birds purchased in those markets. Thus this risk of exposure of people in these countries should be considered too, as has been the case for diseases such as influenza [200,201].

## 12. Concluding Remarks and Future Perspectives

**Figure 5:**
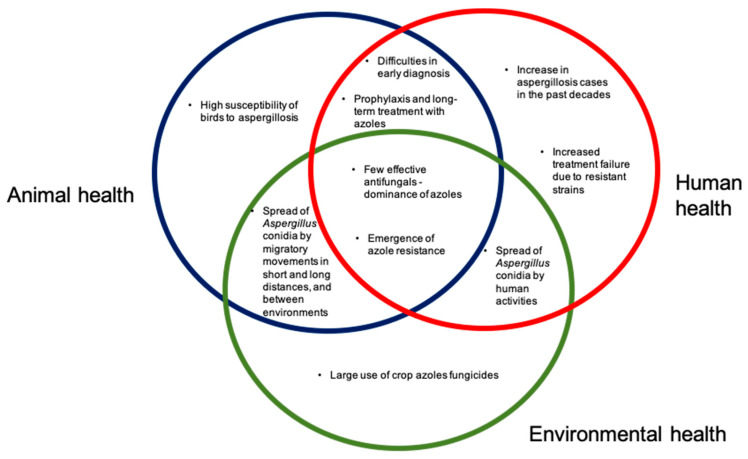
The One Health scheme, demonstrating the implications for *Aspergillus* and aspergillosis, in the environment, in animal health (with emphasis on birds), and in human health.

In summary (Figure 5), there is much evidence suggesting the potential role of avian species in azole resistant *Aspergillus*. Important points, that act as overlapping circles regarding animals, humans and environment, to be highlighted are:

- *Aspergillus* species are ubiquitous fungi, present in many environments, and it is a potential pathogen of importance in animals (we emphasize birds) and humans;
- The emergence of azole resistance in *Aspergillus* species such as *A. fumigatus* sensu stricto is a major concern, limiting treatment success;
- The wide use of crop fungicides has an important role in the emergence of azole resistance;
- Avian species are highly susceptible to aspergillosis. Many species have regular migratory movements, moving between different environments, including those where large amounts of pesticides are used, and natural environments, such as estuaries, lagoons and beaches;
- In their movements between different types of environments, such as agricultural fields, natural environments and urban environments, birds may have an important role in the dispersion of *Aspergillus* isolates, especially resistant strains;
- Considering the characteristics of *Aspergillus* fungi, the importance of aspergillosis in birds and humans, and the emergence of azole resistance, it is essential to promote more investigations in a One Health approach.
- Avian aspergillosis can be used as an indicator of environmental health in specific countries or regions, including surveillance of the introduction of new resistant strains, changes in resistance rates, and emergence of new mechanisms of azole resistance.

After discussing these topics, we suggest some measures that deserve attention in global efforts to overcome the serious and emerging problem of resistance to azoles by *A. fumigatus*, and its consequences to human and animal health, among them:

- Regular cleaning of where birds are kept, with non-azole products, aiming at environmental control of the amount of fungal inoculum;
- Avoid contact of people, in groups at risk for aspergillosis, with poultry farms, zoological institutions, avian wildlife rehabilitation centers;
- Search for more efficient early diagnosis techniques for both humans and birds;
- Implementation of antifungal stewardship programs for both humans and birds;
- Search for new antifungal molecules different from those presently used on crops, with different mechanisms of action, and dissimilar to those used in human therapy;
- Surveillance of antifungal susceptibility in *Aspergillus* strains in environment, birds, and humans;
- Measures to control the dispersion of *Aspergillus* strains in agricultural products transported by humans from farms to urban areas;
- Surveillance of the main routes of bird migration and correlation with spread of azole resistant isolates;
- Search for new fungal control options for fungal control for crops, such as biological rather than chemical control.
